# Supplementary figures and images for: The Aspergillus fumigatus UPR is variably activated across nutrient and host environments and is critical for the establishment of corneal infection
Source: PLoS Pathog. 2023 Oct 31;19(10):e1011435. doi: 10.1371/journal.ppat.1011435 (PMC10637725; doi:10.1371/journal.ppat.1011435)

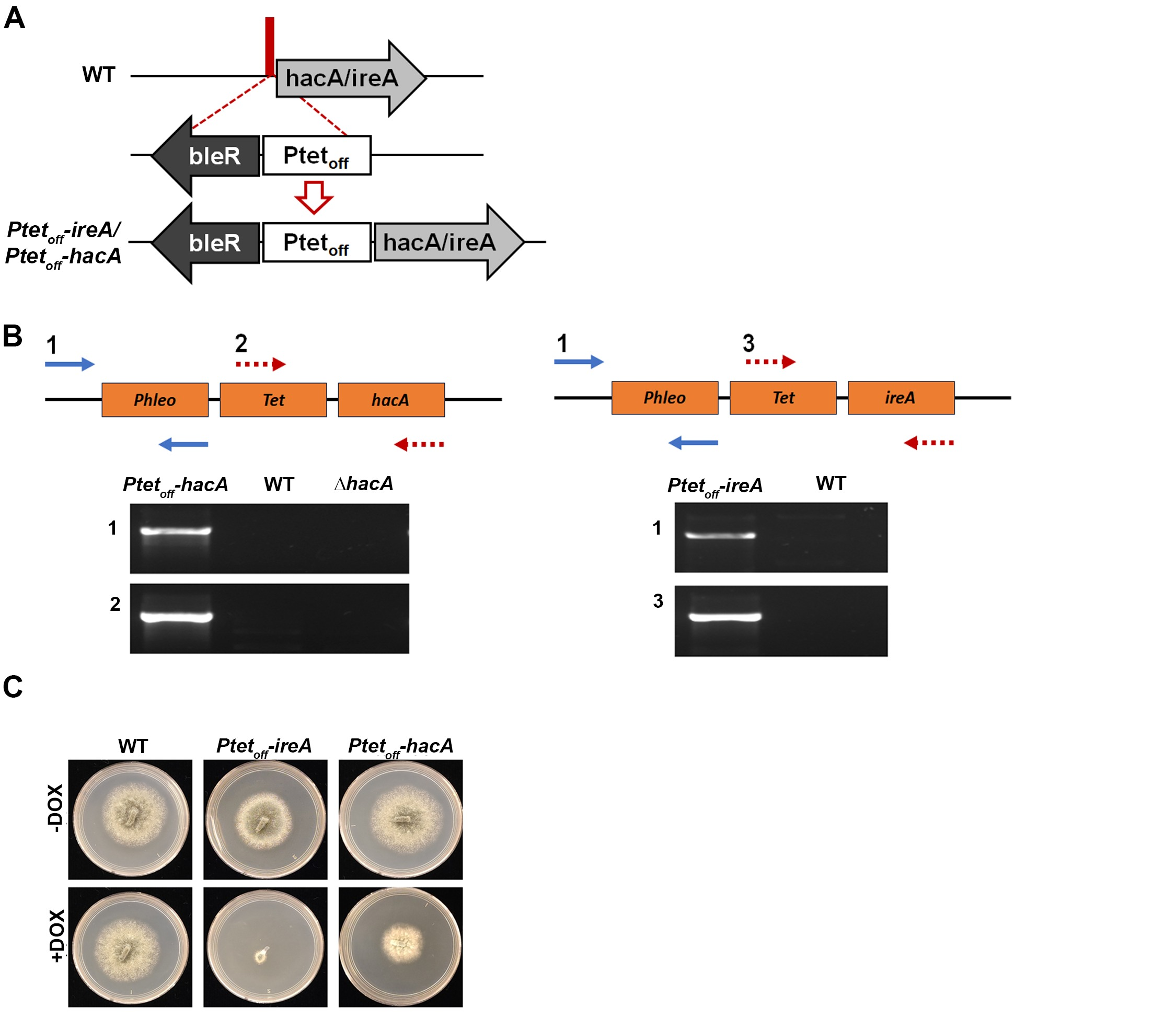

Supplement: S1 Fig — (A) An overview of the hacA deletion strategy using in vitro assembled Cas9 RNPs confirmed by PCR of genomic DNA, (B) 72 h colony diameters of the indicated strains on YPD, GMM with ammonium tartrate as the nitrogen source (GMM-AT), GMM with BSA as the nitrogen source (GMM-BSA), or gelatin minimal medium. Data represented as mean of triplicate samples analyzed by two-way ANOVA **** <0.0001, (C) Colonial appearance of the indicated strains following growth on varying concentrations of brefeldin A (BFA) for 35°C for 48 h. (TIF) [file ppat.1011435.s001.tif]

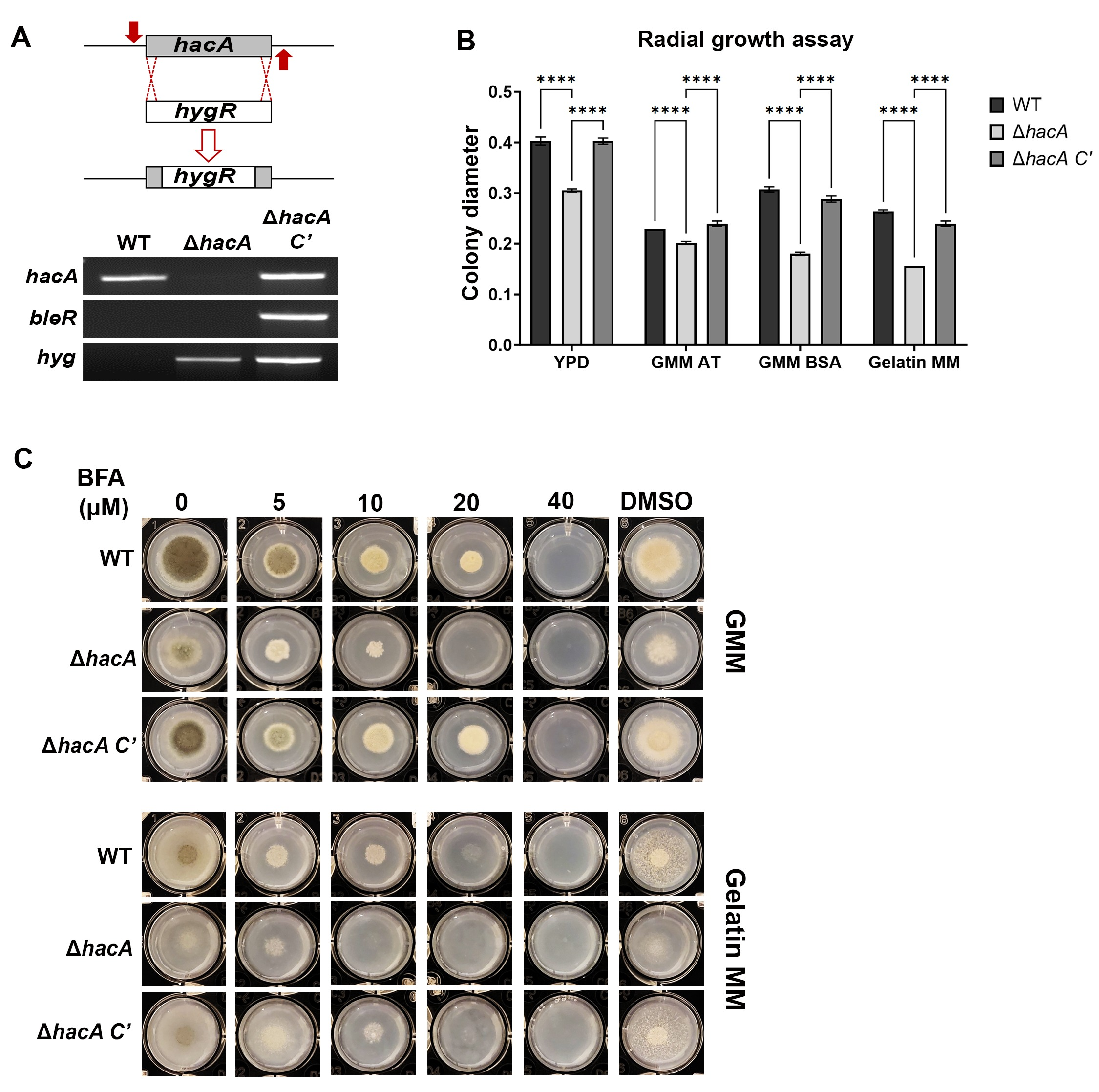

Supplement: S2 Fig — (A) Schematic representation of hacA and ireA promoter replacement with the Tet-Off cassette tagged with the phleomycin resistance gene (bleR), (B) PCR strategy and genotyping of the generated Ptetoff-hacA and Ptetoff-ireA strains, (C) Colonial appearance of the indicated strains grown with or withouth 80 μg/mL doxycycline. Photographs taken following transfer of hyphal plugs onto the media and incubating at 35°C for 72 h. (TIF) [file ppat.1011435.s002.tif]

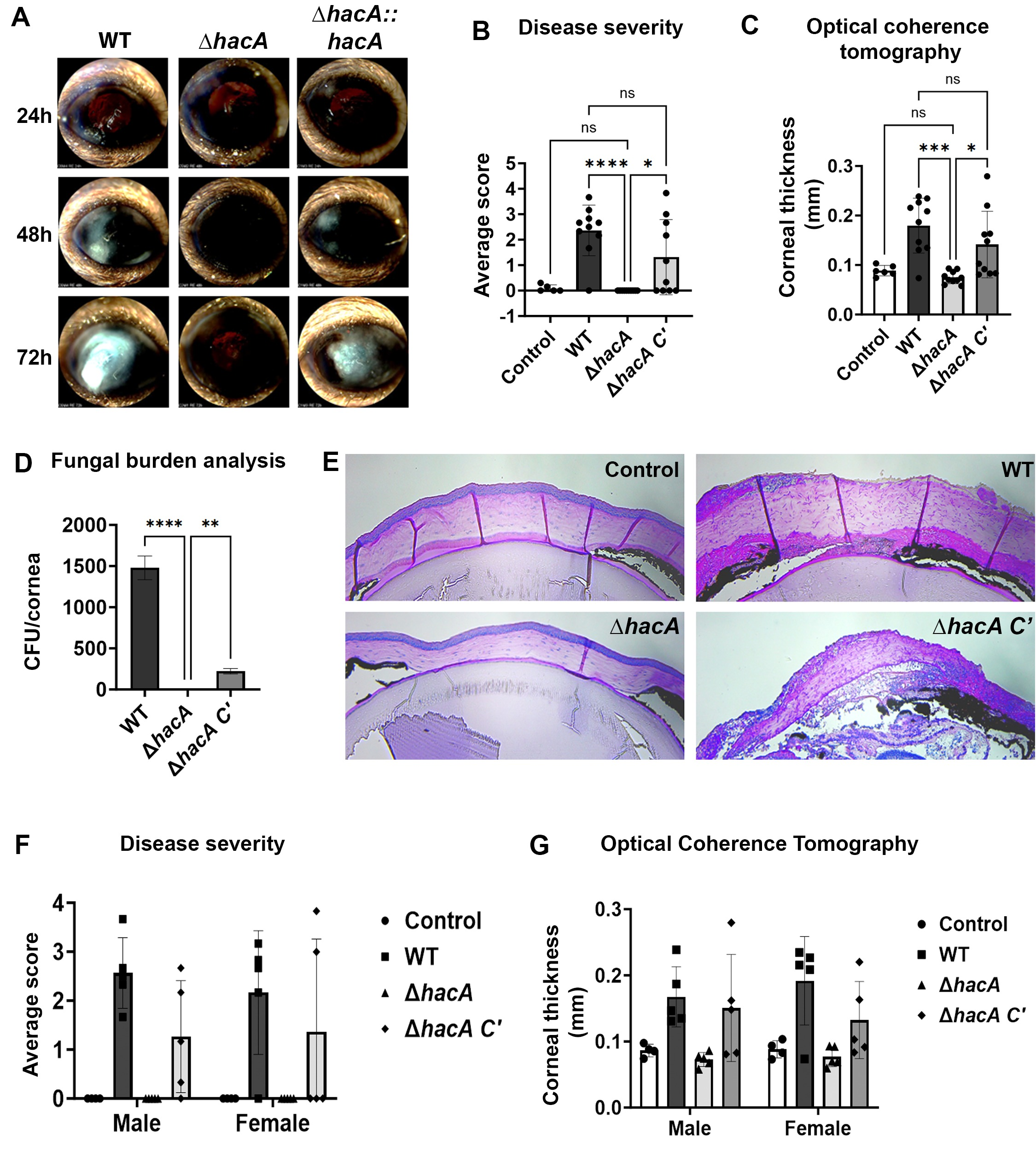

Supplement: S3 Fig — (A) Representative slit-lamp images of infected murine corneas at 24, 48 and 72 h p.i. (B) 72 h clinical disease scores of pooled male and female animals; data analyzed by Ordinary one-way ANOVA **** <0.0001. (C) 72 h corneal thickness measurements based on OCT and measuring 13 points across cornea; (n = 10/group, analyzed by Ordinary one-way ANOVA p-value **** <0.0001. (D) Fungal burden at 72 h p.i. as determined by colony forming unit (CFU) analysis from corneal homogenates; data analyzed by Ordinary one-way ANOVA p-value **** <0.0001; ** 0.0012. (E) Representative PASH stained corneal corneal sections at 72 h p.i. (F) The same 72 h clinical disease scores in panel B with male and female animals separated. (G) The same 72 h corneal thickness measurements in panel C, with male and female animals separated. (TIF) [file ppat.1011435.s003.tif]

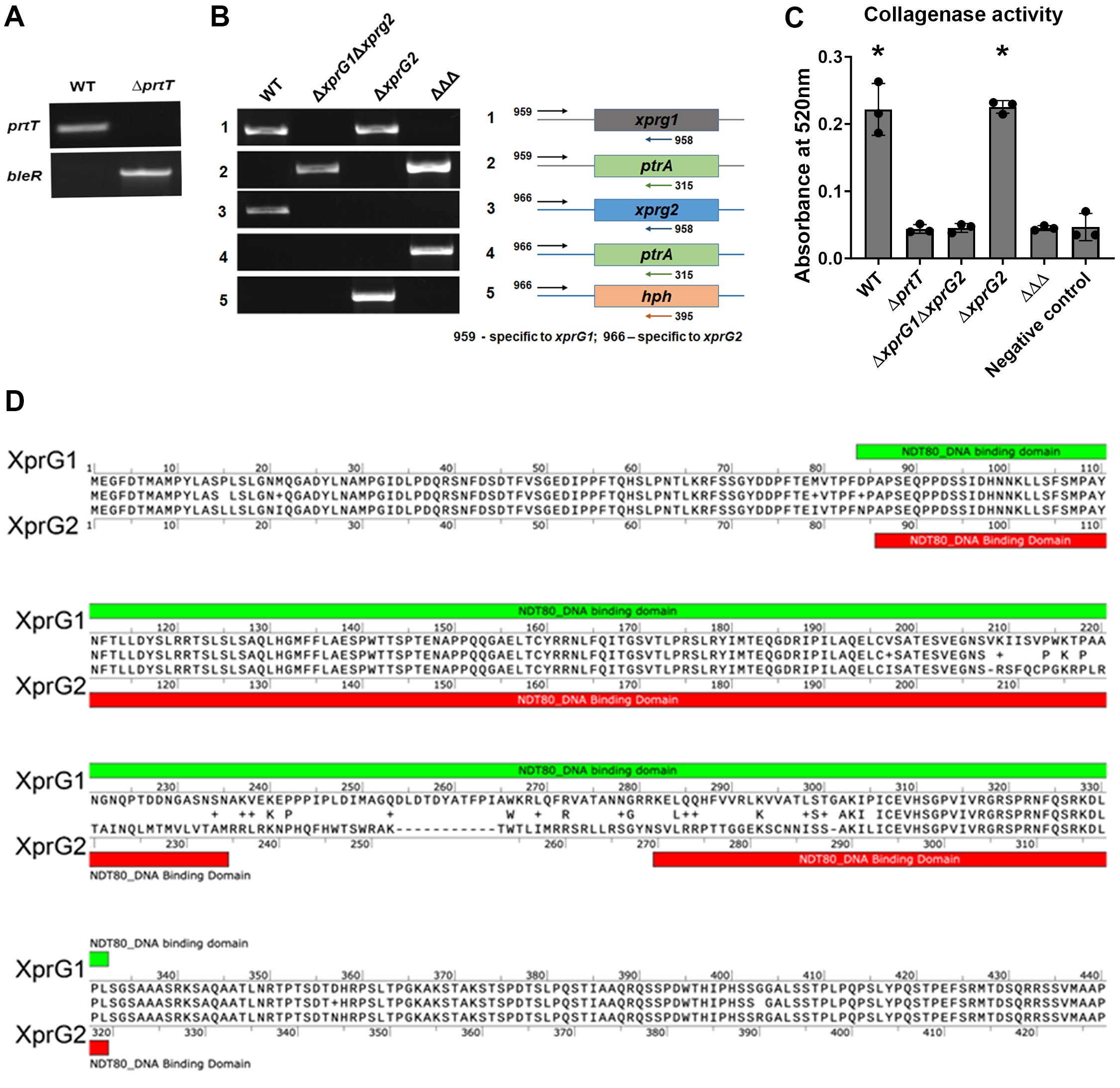

Supplement: S4 Fig — (A) Genomic PCR demonstrating replacement of the prtT coding sequence with the bleR cassette. (B) Genotypic analysis of the ΔxprG1ΔxprG2, ΔxprG2, and ΔprtTΔxprG1ΔxprG2 mutants using a multiple PCR scheme. (C) Collagenase activity of cultured supernatants following 72 h incubation at 35°C in GMM-FBS; data are depicted as raw absorbances at 520 nm following azocoll degradation and represent the mean of triplicate samples analyzed by Ordinary one-way ANOVA * p-value <0.0001 compared to the negative control. (D) Amino Acid sequence alignment for xprG1 (Afu8g04050) and xprG2 (Afu1g00580) using a Smith-Waterman local alignment on SnapGene v7.0.2. The alignment shows NDT80 DNA binding domain (amino acid residues 86–290) in XprG1 (green) and the putatively non-functional NDT80 (missing amino acid residues 235–270) domain of XprG2 sequence (red). (TIF) [file ppat.1011435.s004.tif]

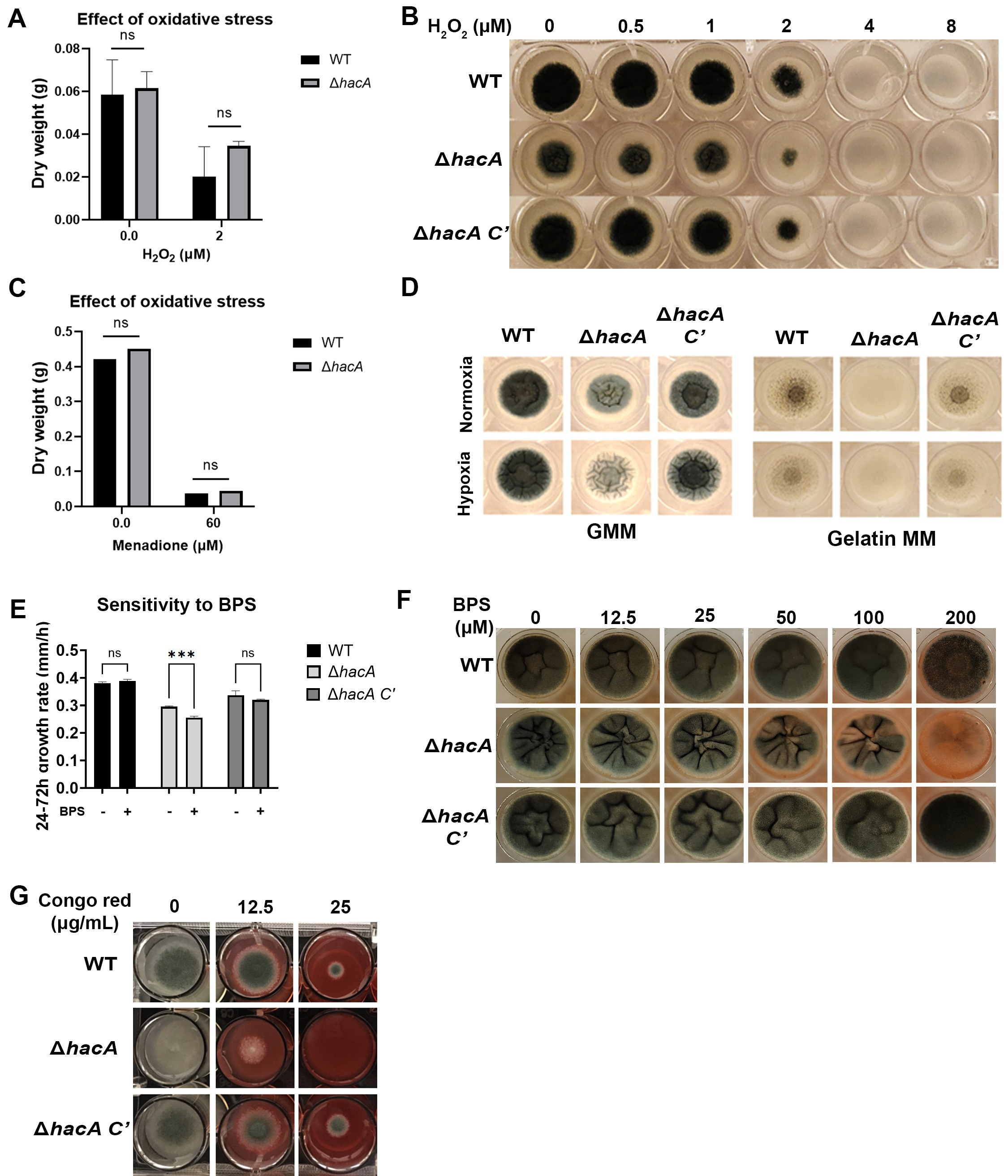

Supplement: S5 Fig — (A) Dry weight of Af293 WT and ΔhacA grown in liquid GMM with and without 2 μM H2O2 for 72 h at 35°C; data represent the mean of triplicate samples analyzed by unpaired T-test. (B) Conidia of WT and ΔhacA were spotted on GMM containing a concentration gradient of H2O2. Photographs taken after 72 h incubation at 35°C. (C) Dry weight of Af293 WT and ΔhacA grown in liquid GMM with and without 60 μM menadione for 72 h at 35°C; data represent the mean of triplicate samples analyzed by unpaired T-test. (D) Photographs taken of the indicated strains following growth on GMM and gelatin MM at 35°C for 72 h in normoxia or hypoxia (1% O2). The ΔhacA mutant is developmentally delayed on gelatin, but its mycelial growth is indistinguishable in normoxia and hypoxia. (E) 24–72 h growth rate measurement on YPD plates with 200 μM of the iron chelator bathophenanthrolinedisulfonate (BPS). (F) Conidia of the indicated strains were spotted onto GMM a concentration gradient of BPS; photographs taken after 72 h incubation at 35°C. (G) Conidia of the indicated strains were spotted onto GMM a concentration gradient of Congo red; photographs taken after 72 h incubation at 35°C. (TIF) [file ppat.1011435.s005.tif]
