# Supplementary material for: The Aspergillus fumigatus UPR is variably activated across nutrient and host environments and is critical for the establishment of corneal infection
Source: PLoS Pathog. 2023 Oct 31;19(10):e1011435. doi: 10.1371/journal.ppat.1011435 (PMC10637725; doi:10.1371/journal.ppat.1011435)
Supplement: Attachment — Submitted filename: Response to reviewer comments.docx [file ppat.1011435.s015.docx]

**Response to reviewer comments**

***Summary***: We thank the editor and reviewers for their time and insightful evaluation of our manuscript. In response to effectively all comments, we have added new experimental data and/ or altered the prose, which we feel has altogether broadened and strengthened this story. In some cases, additional experiments were tried but did not yield interpretable results, and those are discussed below in our point-by-point response where appropriate.

**Reviewer #1:**

Major comments

1. *The sterilization of the mutant within 72 contrasts the attenuated virulence of this mutant in a model of invasive pulmonary aspergillosis, suggesting an important tissue-specific virulence defect. However, an alternate explanation is that the difference is due to variations in the genetic background of the fungus and/or the use of more recent technology for gene deletion (the IPA study was published in 2009). An experiment showing that the newer mutant developed in this study can still cause disease in a pulmonary model would clearly demonstrate that UPR support of corneal infection is context-specific and would strengthen the manuscript*.

This is an excellent point that we have addressed with new experiments. We first attempted to run the IPA experiment under conditions that closely reflects our corneal model (C57BL/6 mice + methylprednisolone), but none of the WT-inoculated animals developed the disease. While this could be solely due to an inadequacy of methylpred as an immunosuppressant in this model, we reasoned the weaker (and well-documented) virulence of Af293 was the key issue. We then turned to a model that more closely reflected the previously published work (Richie et al., 2009), which included outbred mice (CD-1 in our case) and triamcinolone injections. In this model, we observed sufficient disease development and mortality in the WT and complementer groups, but effectively no morbidity and mortality in the *∆hacA* group. This avirulent phenotype of the Af293 *∆hacA* does indeed differ from the hypovirulent phenotype of the AfS28 strain (Richie et al.), which we interpret as another example of strain heterogeneity. These results are now included as primary data in the manuscript (Figure 5) and are discussed accordingly.

We also analyzed *hacA* splicing from infected lung tissue. Surprising, and in contrast to our cornea data, we found the *hacA^i^/hacA^u^* splice ratio was similar to *in vitro* samples treated with DTT. Thus, we conclude that the ER stress experienced by the fungus does indeed differ across host environments, which leads to varying, but nevertheless essential, HacA readouts (based on the RNA-seq data). This is now a central theme of the discussion and is furthermore reflected in the title change.

Altogether, we believe the inclusion of the lung virulence and splicing data have considerably strengthened our story and we thank the reviewers for setting us on this path.

2. *Another explanation for the inability of the mutant to infect the cornea is that it is hypersensitive to antimicrobial compounds in tear film (for example, the hacA mutant in Alternaria is hypersensitive to plant antimicrobial compounds). Does the hacA mutant show hypersensitivity to any of the major antimicrobial compounds found in tear film?*

We think this is another great point and we performed experiments with two antimicrobial peptides that we reasoned would yield promising results. The first was lysozyme, which hydrolyzes polysaccharides of the bacterial and fungal cell wall and is abundant in the tear film. Given the demonstrated cell wall phenotype of *∆hacA*, we reasoned the mutant would be hypersensitive to lysozyme-mediated killing. We did not, however, find a concentration of protein or media condition in which lysozyme inhibitory to the WT *A. fumigatus*, and no relative difference of the *∆hacA* was observed. We still think this is an important line of investigation and will continue to expand and optimize our experimental parameters, but for now they remain undiscussed in the manuscript.

The second compound was with the iron chelating protein lactoferrin based, which we reasoned would be important based on two lines of evidence: (1) The Pearlman group demonstrated topical treatment with lactoferrin is therapeutic during FK that the *A. fumigatus* ∆*sidA* mutant is attenuated for virulence in the model; (2) the Askew group demonstrated their *∆hacA* mutant is hypersensitive to the iron chelator BPS. We therefore hypothesized that our mutant would show altered growth in the presence of lactoferrin, which would contribute to its avirulent phenotype in our model. Similar to the lysozyme experiments, however, and despite trying variety of media conditions (including low iron media) and lactoferrin concentrations, we did not observe any degree of inhibition in the WT strain, nor did we observe differences between WT and *∆hacA*. While we cannot directly conclude the mutant is hypersensitive to lactoferrin in particular, we did however demonstrate a hypersensitivity to BPS, which is now included in the supplementary data. Hopefully, our discussion about iron chelation and its potential influence in the virulence of *∆hacA* (beginning on line 532 of the unmarked document) will suffice while we continue to optimize our experiments.

3. ***Line 353***. *“to our knowledge, there has been no direct assessment of whether any fungal pathogen activates the UPR in host tissue”. This should be corrected since activation of the UPR in vivo has been shown at least for cib1 (hacA homolog) in Ustilago Maydis (Plant cell 2010, mBio 2019). Since hacA is not induced in the cornea, this would support a model in which the impact of the UPR on virulence is tissue-specific, which is interesting and has important implication for future therapies that target the pathway*.

We thank the reviewer for their insight and have included the *Ustilago* story in our discussion (line 562 in the unmarked document). Of course, we also agree with the comment about the tissue-specific contributions of the UPR, which we have attempted to address with our assessment of virulence and *hacA* splicing in the lung model (described above).

Minor comments

*For the nutritional experiments, was the medium solidified with agar or agarose (this should be mentioned in the methods)? Agar provides trace nutrients that can confound nutritional experiments. This is not a major point, since there is a clear difference with the mutant regardless, but the authors may see a bigger difference if agarose is used.*

We thank the reviewer for pointing out this oversight and we have now indicated that that agar was used as the solidifying agent in our experiments (line 594 of the unmarked document). To the reviewer’s second point, we have indeed demonstrated that the relative growth differences of *∆hacA* on proteinaceous substrates are more marked when agarose is used instead. Since our various compound stressors (e.g. BFA or Congo red) were performed on agar media, and towards not re-running all of those assays on agarose, we have decided to display our agar media results across the board for the sake of continuity.

***Line 20*** *– ‘completely’ is redundant with ‘unable’*

Quite right. This has been corrected.

***Line 163*** *– consider adding language to explain the difference between in vivo and in vitro baseline. i.e. “…beyond baseline, defined as in vitro conditions where the medium supplies reduced sources of carbon and nitrogen that do not require breakdown by secreted fungal hydrolases”.*

We thank the reviewer for pointing out our vague description of what is indeed a critical aspect of our experimental design. We have added the clarifying language as suggested (line 172 of the unmarked document).

***Line 263****. Fig. 1 should be Fig. 3*

We apologize for the oversight. The figure numbering has been grossly altered in the revised manuscript and we believe everything is in order now.

***Line 421****. Missing reference (Pearlman paper)*

We apologize for the oversight and have now added the appropriate reference.

*Since many outside the field are not familiar with this model, I think it would be worthwhile to clarify early in the manuscript that the model creates an epithelial breach to allow the fungus to colonize the collagen-rich environment of the corneal stroma. I also think it would be worth emphasizing that the cornea is a unique environment relative to the lung, particularly in the context of vascularization and immune-privilege (which could account for why the findings for this mutant differ between cornea and lung).*

Thank you for these suggestions. Regarding the details of our model, we hope the reviewer agrees that that this is sufficiently addressed early in our results (beginning at line 150 in the unmarked document). We have also added a discussion of the corneal environment in our introduction (beginning at line 120). The idea that virulence of the mutant may vary between the lung and cornea as a function of the immune response is a good one, and we feel this has been addressed in our discussion of the newly added lung experiments.

**Reviewer #2:**

Major comments

1. *The primary inferences in the manuscript are based on the RNA data as investigated by transcriptomics or RT-PCR, or qRT-PCR. However, transcriptomic studies only provide a snapshot of RNA levels at a given time point. Thus, mRNA dynamics may not correlate with protein levels.*

We appreciate this comment. While we haven’t addressed alterations in protein levels experimentally, we have included a discussion of how the UPR may be regulated at the post-transcriptional and post-translational level (beginning around line 441 in the unmarked document).

2. *Authors argue that bUPR and iUPR may represent two ends of the UPR spectrum, and their corneal model is a source of sub-acute ER stress. This was not shown with changes in Hac-induced levels that were similar to basal levels. This was inferred based on changes in the transcriptome, which suffers from the argument in point 1.*

Yes, we have to concede that this issue of steady-state HacA protein levels across different environments remains an important line of experimentation not addressed here. Indeed, our ongoing work includes generating tagged *hacA* alleles that will allow for the analysis of protein levels as well as HacA binding to its downstream targets. For now, we hope our discussion of post-transcriptional regulation (described above) will serve as an acceptable place holder for these data.

3. *Authors have mainly argued hacA-induced levels as the sub-acute level of ER stress. However, they fail to discuss the hacA independent role(s) of sensor ireA (PMID 22028661). How does basal and/or subacute ER stress (if it exists) regulate ireA and hacA independent functions?*

The question of how IreA may be fine-tuning the adaptive response to these various environments is very interesting and one we have attempted to address with new experiments. We began by trying to generate an *ireA* deletion mutant, but were surprised and frustrated by the fact that all of our transformants (from Cas9 or standard homologous recombination strategies) retained the gene. This led to the hypothesis that *ireA* may be essential in Af293 even under nominally homeostatic conditions, which is in agreement with our private corresponded with Dr. Jarrod Fortwendel lab (at UTHSC) who similarly could not isolate a *∆ireA* strain during their construction of a kinase-deletion library (CEA10 background). To confirm this was a biological issue (i.e. the gene is essential) and not a technical one, we next generated strains in which the *ireA* and *hacA* genes was placed under the control of a doxycycline-repressible promoter. We observed that doxycycline supplementation of the *tet-hacA* strain resulted in a phenotype that was comparable to *∆hacA*, whereas the *tet-ireA* strain demonstrated almost no hyphal growth. This is consistent with the “essentiality” hypothesis and this work is now thoroughly described in the manuscript and presented as supplemental data.

We recognize, of course, that these results do not strictly address the original question (what is IreA doing?!) and we are following up with our *tet-ireA* system to address this issue through transcriptomics and other analyses. For this manuscript, however, we have decided to limit our experimental data and the accompanying discussion to HacA output and its possible regulation by IreA-independent mechanisms. We hope the reviewer finds this this acceptable as we further explore IreA for a follow-up paper.

4. *Also, there is a direct link between the calcineurin pathway and ER stress response in baker’s yeast. How calcineurin and other known pathways of ER stress play a role are not mentioned/discussed.*

We appreciate the reviewer’s insight on this matter and have added a discussion that addresses the calcineurin-UPR connection established in *A. fumigatus* (beginning line 452 of the unmarked document). More completely, we postulate that the loss of *∆hacA* results in calcium dysregulation and activation of the calcineurin pathway; consequently, the downstream activity of calcineurin-regulated transcription factors, such as CrzA, could be contributing to the transcriptomic changes we observe in the *∆hacA* mutant.

5. *Another major argument is the increased expression of major collagenase genes (Fig1b). How is that related to virulence? A ΔprtT mutant deficient in the secretion of these major proteases showed ex vivo defects but was indispensable for virulence. If the changes in the secretory pathway induce ER stress that is essential for infection and if these major proteases are involved in infection, authors should discuss the prtT data (PMID 19564385). Additionally, as glucose supplementation did not change corneal infection, the role of Er stress-dependent protease expression is not clearly defined.*

The reviewer has raised several important points here. Our initial hypothesis was based on the idea that increased expression of proteases *in vivo* would lead to secretory/ER stress, followed then a discernable increase in *hacA* splicing and UPR activation. We have attempted to clarify this rationale in the prose (beginning on line 145 in the unmarked document). To the reviewer’s next point, our data raise the question of what role, if any, do these proteases play in virulence. Stated another way: does a marked reduction in protease activity contribute to the virulence defect of *∆hacA*? To address this, we generated our own *prtT* deletion mutant as well as mutant deficient in both *prtT* and another protease transcription factor *xprG1*. We demonstrate that (1) these mutants display a loss of detectable protease activity, as expected, and (2) the mutants retain full virulence in our model of FK. Thus, we conclude that the loss in protease activity is NOT the key determinant of the *∆hacA* virulence defect. These data are included as a primary figure (Figure 4).

Regarding the glucose supplementation experiment: we have actually removed those data from the manuscript in response to a point raised by reviewer #3 (see below). But for the sake of discussion, we hypothesis that the presence or protein (not necessarily the lack of soluble macronutrients) is sufficient to drive increased hydrolase expression. Even if those hydrolases are not strictly required for growth in that environment, their production nevertheless induces ER stress that the *∆hacA* cannot deal. In this way, the avirulence of the UPR mutant is still tied to nutritional environment, but not necessarily a lack of nutritional acquisition. This likely doesn’t make sense, but we are following up in it!

6. *As the authors discussed the multifactorial role(s) of hacA in nutrient stress, secondary metabolism, and other growth features (cell wall homeostasis), authors need to take into account that hacA mutant showed 50% mortality compared to the control. Thus, their aim of finding inhibitors that can be used to treat both IPA and FK still needs more work. As these survival/mortality analyses differ with the immune suppression, the major secondary metabolite gliotoxin might be playing a role; however, the authors fail to discuss the role(s) of immune suppression.*

These are good points. We believe we have largely addressed the “lung v cornea” issue through the inclusion of data that show our *∆hacA* mutant is also avirulent in a model IPA. This does indicate that the important of HacA is variable between strains, so HacA inhibitors would also display variable clinical usefulness. We would argue that this is true for essentially any putative drug target in any organism! Feng et al. demonstrated that the *ireA* mutant is essential for virulence during IPA and we show it is essential altogether in strain Af293. Thus, the IreA protein is likely a better candidate as a therapeutic target and we have been intensively exploring IreA inhibitors in a treatment model of FK. This manuscript is currently in preparation and we look forward to including the IPA model in the study.

The note about gliotoxin’s contribution to virulence as a function of the immune status of the host is an excellent one. Accordingly, we have included a new section in the discussion (beginning at line 542 in the unmarked document) that addresses this idea with respect to HacA’s apparent regulation of secondary metabolism based on our RNA-seq data.

7. *Authors should be careful in drafting the manuscript. Line 421, “Pearlman paper,” should be properly cited.*

We apologize for the oversight and have now added the appropriate reference.

Minor comments

*Please superscript the divalent metal cations. The degree sign seems to be a superscript "O" and not the degree symbol.*

We apologize for the oversight on these formatting issues and they have corrected them accordingly.

**Reviewer #3:**

Major comments

1. *Some titles are contrary to the data presented e.g. Transcriptomics supports distinct roles for the UPR under acute ER and nutritional stress. In this section the transcriptomic data does not seem to support any major role for the UPR in ‘nutritional stress’. Although I do accept there is a role for UPR during growth in nutritional stress (as there is for growth per se)– the requirement for hacA on proteinaceous media is not presented until much later in the manuscript.*

We agree with the reviewer on this point and have now consolidated all our RNA-seq together (WT and *∆hacA*) at the end of the results section. We think this works much better.

2. *Line 239 states that secretion of collagenases was impaired in the mutant – at that point in the manuscript the authors had not established that loss of hacA was not involved in transcriptional regulation of these enzymes – and hence a conclusion could be drawn that production (not just secretion) of collagenases was affected.*

This is indeed a fair point and we have qualified that statement accordingly (line 215 of the unmarked document).

3. *The link between the requirement for protease activity and investigation of the UPR is not well made following the first results section.*

We thank the reviewer for this comment, which mirrors to some degree a point raised by reviewer #2. Our initial hypothesis was based on the idea that increased expression of proteases *in vivo* would lead to secretory/ER stress, followed then a discernable increase in *hacA* splicing and UPR activation. We have attempted to clarify this rationale in the prose (beginning on line 145 in the unmarked document).

A few other critical issues with the ms:

1. ***Table 1****: the legend lacks any meaningful description of the data presented. Also Exact p-values are presented: Given the dataset presented it is more appropriate to present corrected p-values (Benjamini-Hochberg) to account for false discovery. I suspect that this will remove protein folding as a hit from the DTT data however it is sufficient just to state the specific genes affected in this group to show impact on protein folding.*

We thank the reviewer for this comment. The terms included in the table were actually based on adjusted p-value cut-offs. However, we transcribed the exact p-values in error. So, while we have corrected the p-values accordingly, the terms included actually did not change (including the protein folding category). We further apologize for the original Table legend/title; this has been corrected.

2. *The supplementation of nutrients in the animal infection model with glucose does not account for the fact that Af is probably using the proteinaceous component of the cornea as a nitrogen source. This should be addressed.*

We appreciate this insightful critique. We have unpublished data that shows glucose supplementation to the cornea can rescue the virulence defect of an *A. fumigatus* mutant that has a carbon metabolism defect. We reasoned that if we could similarly rescue the virulence defect of *∆hacA*, then we could make the case that the loss of virulence is tied to reduced carbon assimilation. As we didn’t observe such as rescue, and to the reviewer’s point, we are still left to consider the role of nitrogen assimilation. As these experiments are ongoing, we have decided to omit the glucose experiment from the manuscript.

*Overall I feel the narrative and logical flow of the manuscript needs addressing. This will require reordering of the data presented and highlighting the critical points of the work more clearly.*

The reviewer will find that the integration of new experimental figures, and in response to some concerns raised by the other reviewers, has forced us to fundamentally rework the story. As a result, we believe the flow is much more logical.

Minor comments

*Here seems to be some inappropriate self-citation on line 224 – Lightfoot and Fuller seems to refer to transformation of Fusarium solani.*

This is a fair comment and the reference has been removed.

*The legend for figure 1 is not sufficiently detailed. Panel (B) – assume the transcripts have been normalised to B-tubulin and then compared to growth in standard culture conditions? Panel D include timepoints.*

We appreciated these critiques. We have overhauled all of our figure legends and believe all issues have been addressed.

***Figure 2*** *is not contributing anything to the paper – remove.*

This is definitely fair. We have replaced that figure with a new Venn diagram (Figure 9) that summarized the correspondence of *∆hacA* DEGS across the various conditions. We believe this is much more informative and germane to our central story.

***Figure 3*** *E- legend lacking detail.*

Thank you. We have heavily edited all of our figure legends and believe all issues have been corrected.

*The references are numbered at the end of the document but named in the ms- this made review difficult.*

We apologize for this oversight and have corrected the issue.

*Figure title for 7 is not appropriate.*

We apologize for this issue. As those data have been removed from the manuscript, the issue has corrected itself!

***Line 421*** *– ‘Pearlman paper’ needs reference.*

We apologize for the oversight and have now added the appropriate reference.

*All figure legends should be checked carefully to ensure the data is correctly described.*

Thank you. We have heavily edited all of our figure legends and believe all issues have been corrected.
